# Supplementary material for: The importance of active surveillance of carbapenem-resistant Enterobacterales (CRE) in colonization rates in critically ill patients
Source: PLoS One. 2022 Jan 20;17(1):e0262554. doi: 10.1371/journal.pone.0262554 (PMC8775193; doi:10.1371/journal.pone.0262554)
Supplement: Attachment — Submitted filename: Response to Reviewers.docx [file pone.0262554.s001.docx]

**Rebuttal Letter**

**In response to the reviewers’ comments to the Author**

**Reviewer #1:** The Aims and objectives to be clearly defined

Line 94 and 138- enterobacteria is it a standard terminology it must be Enterobacteriaceae

**Answer Line 94:** I did not identify the enterobacteria word at line 94.

**Answer Line 138:** enterobacteria was replaced by “**Enterobacteriaceae**”.

Invasive infections were found be more in colonized patients than the control group, is it after application of all the preventive measures as per the hospital policy.

**Answer:** Yes, invasive infections in colonized patients occurred after application of all the preventive measures.

**Line 228 and 231-** to be written as association of risk factors and colonization not influence needs statistical consultation for using these terminologies

**Answer:** It’s correct! The terminology was replaced after consult the statistical books.

**Reviewer #2:** The manuscript highlights an important problem of gut colonization with CRE in a high risk population and I wish the best to the authors.

**Answer:** Thanks!

**Correction the following review comments:**

1) Carbapenem resistant Enterobacteriaceae (CRE) should be replaced with "CR Enterobacterales" at all places including the title

**Answer:** "CR Enterobacterales" was replaced at all places including the title

2) Pg 5: study design, line 115: The method of processing the rectal swab must be elaborated for the benefit of the readers along with a reference

**Answer pg 5: study design, line 115:** The method of processing the rectal swab was written of the clear form on page 6: data collection, line 151, along with a reference.

3) Pg 6: data collection, line 130 mentions data collection from 2012 to 2018. Then Line 133: mention data included from 2014 to 2018. Please mention clearly the duration of the study

**Answer pg 6: data collection, line 130 and 133:** The duration of the study was mentioned for best comprehension of the readers.

4) No clarity on the following issues:

• Whether patients were kept in contact isolation till results arrived?

**Answer:** No, the patients weren’t kept in contact isolation till the results arrived. Therefore, the phrase of the pg. 5: study design, line 122 was reformulated for best comprehension the readers.

• What was the turnaround time of the CRE surveillance results?

**Answer:** The turnaround time mean of the CRE surveillance results was of five days. These data were described on pg. 7: data collection, line 154, for best comprehension of the readers.

• What media were used for surveillance purposes?

**Answer:** The media used for surveillance purposes were information immediate of the microbiology laboratory and notifications in the electronic record and prescription medical by Hospital Infection Control Sector. These data were described on pg. 7: data collection, line 155, for best comprehension of the readers.
