# Supplementary material for: The importance of active surveillance of carbapenem-resistant Enterobacterales (CRE) in colonization rates in critically ill patients
Source: PLoS One. 2022 Jan 20;17(1):e0262554. doi: 10.1371/journal.pone.0262554 (PMC8775193; doi:10.1371/journal.pone.0262554)
Supplement: Attachment — Submitted filename: Response to Reviewers.docx [file pone.0262554.s002.docx]

**Rebuttal Letter**

**In response to the reviewers’ comments to the Author**

**1.** If the authors have adequately addressed your comments raised in a previous round of review and you feel that this manuscript is now acceptable for publication, you may indicate that here to bypass the “Comments to the Author” section, enter your conflict-of-interest statement in the “Confidential to Editor” section, and submit your "Accept" recommendation.

**Reviewer #1:** All comments have been addressed

**Reviewer #2:** All comments have been addressed

**2.** Is the manuscript technically sound, and do the data support the conclusions?

The manuscript must describe a technically sound piece of scientific research with data that supports the conclusions. Experiments must have been conducted rigorously, with appropriate controls, replication, and sample sizes. The conclusions must be drawn appropriately based on the data presented.

**Reviewer #1:** Yes

**Reviewer #2:** Yes

**3.** Has the statistical analysis been performed appropriately and rigorously?

**Reviewer #1:** Yes

**Reviewer #2:** Yes

**4.** Have the authors made all data underlying the findings in their manuscript fully available?

The [PLOS Data policy](http://www.plosone.org/static/policies.action#sharing) requires authors to make all data underlying the findings described in their manuscript fully available without restriction, with rare exception (please refer to the Data Availability Statement in the manuscript PDF file). The data should be provided as part of the manuscript or its supporting information or deposited to a public repository. For example, in addition to summary statistics, the data points behind means, medians and variance measures should be available. If there are restrictions on publicly sharing data—e.g. participant privacy or use of data from a third party—those must be specified.

**Reviewer #1:** Yes

**Reviewer #2:** Yes

**5.** Is the manuscript presented in an intelligible fashion and written in standard English?

PLOS ONE does not copyedit accepted manuscripts, so the language in submitted articles must be clear, correct, and unambiguous. Any typographical or grammatical errors should be corrected at revision, so please note any specific errors here.

**Reviewer #1:** Yes

**Reviewer #2:** Yes

**6.** Review Comments to the Author

Please use the space provided to explain your answers to the questions above. You may also include additional comments for the author, including concerns about dual publication, research ethics, or publication ethics. (Please upload your review as an attachment if it exceeds 20,000 characters)

**Reviewer #1:** All the comments are addressed and incorporated in the manuscript. The study is an important research work adds to the medical literature the relevance of looking for multi drug resistant bacterial colonizers and implement preventive measures to control their spread in the critical areas of the health settings

**Answer:** Thanks your comments!

**Reviewer #2:** Thank you for clarifying the comments. Pls find below the comments from the fresh review

1) Pg 66, line 73: Capital E for Enterobacterales

**Answer Line 73:** enterobacterales was replaced by “**Enterobacterales**”.

2) Pg 69 line 141: pls change to Enterobacterales

**Answer Line 138:** *Enterobacteriaceae* was replaced by “**Enterobacterales**”.

7. PLOS authors have the option to publish the peer review history of their article ([what does this mean?](https://journals.plos.org/plosone/s/editorial-and-peer-review-process#loc-peer-review-history)). If published, this will include your full peer review and any attached files.


If you choose “no”, your identity will remain anonymous but your review may still be made public.


**Do you want your identity to be public for this peer review?** For information about this choice, including consent withdrawal, please see our [Privacy Policy](https://www.plos.org/privacy-policy).

**Reviewer #1:** No

**Answer:** Thanks your review in this article!

**Reviewer #2:** **Yes:** Aruna Poojary

**Answer:** Thanks your review in this article!
